# Impact of an enhanced anti-infection prophylaxis strategy for pancreatoduodenectomy: a single centre analysis

**DOI:** 10.1007/s00423-024-03465-y

**Published:** 2024-10-15

**Authors:** Tina Groß, Felix Merboth, Anna Klimowa, Christoph Kahlert, Marius Distler, Jürgen Weitz, Thilo Welsch, Benjamin Müssle

**Affiliations:** 1 Department of General, Visceral and Thoracic Surgery, University Hospital Hamburg-Ependorf, Hamburg, Germany; 2 Department of Visceral, Thoracic and Vascular Surgery, University Hospital Carl Gustav Carus, Technische Universität Dresden, Dresden, Germany; 3 National Center for Tumor Diseases (NCT/UCC), Dresden, Germany; 4 Department of General, Visceral and Transplantation Surgery, University Hospital Heidberg, Heidelberg, Germany; 5 Medical Faculty, Technische Universität Dresden, Dresden, Germany; 6 Present Address: Department of General and Visceral Surgery, University Hospital Ulm, Ulm, Germany

## Abstract

**Introduction:**

Surgical site infection (SSI) after pancreatoduodenectomy (PD) is a significant concern. Targeted antibiotic prophylaxis (pAP) has been tested to mitigate antibiotic resistance patterns, especially after preoperative bile duct stenting. The aim of this study was to investigate the effect of enhanced anti-infective prophylaxis (EAP) on the incidence of superficial and intraabdominal SSI.

**Methods:**

All patients who underwent PD at a single centre between May 2018 and May 2021 were retrospectively analysed. A control cohort of patients who received pAP with intravenous cefuroxime and metronidazole and routine intraoperative abdominal lavage according to the surgeons’ preferences. Since March 2020, pAP has been changed to piperacillin/tazobactam according to local resistance patterns and combined with routine intraoperative extended abdominal lavage (EIPL). Preoperative selective decontamination of the digestive tract (SDD) has been applied routinely since Jan 2019.

**Results:**

In total, 163 patients were included. The standard (*n* = 100) and EAP (*n* = 63) groups did not significantly differ with regard to pertinent patient and operative characteristics. In the EAP group, the rates of SSI (14% vs. 37%, *p* = 0.002, total rate: 28%) and urinary tract infection (24% vs. 8%, *p* = 0.011, total rate 18%) were significantly lower. Other septic complications were not significantly different. In addition, the risk of developing gastrointestinal bleeding and delayed gastric emptying was significantly lower in the EAP group. Multivariate analysis showed that an age > 67 years was a significant risk factor for SSI.

**Conclusion:**

The results indicate that enhanced anti-infective prophylaxis may significantly decrease the incidence of SSI in patients after PD.

## Introduction

Pancreatoduodenectomy (PD) is the only curative treatment option for patients with pancreatic head, distal bile duct, or ampullary tumours [1–4]. Although the outcomes of patients who underwent PD have improved significantly through advances in surgical techniques and increasing centralization at high-volume centres, PD remains a complex procedure with a relatively high morbidity rate of 30–60% [4–6]. The morbidity rate is sustained through postoperative complications such as postoperative pancreatic fistula (POPF), postpancreatectomy haemorrhage (PPH), delayed gastric emptying (DGE), and surgical site infections (SSI), which occur in almost half of the patients [1, 3–5, 7].

SSI increases the length of hospital stay, reoperation rate, and number of in-hospital deaths, thus constituting a significant negative impact on the postoperative outcome of PD [2, 3, 8]. In addition, SSI also increases health care costs [2, 5, 6]. According to the literature, the rate of SSI after abdominal surgery is generally 2–5%. In contrast, the rate of SSI in patients who underwent pancreatic surgery accounts for 10–30% [9–11]. In the presence of SSI, the hospital stay is prolonged by approximately 10 days [8]. Therefore, the prevention of SSI in patients undergoing PD represents a key challenge in the perioperative management of these patients.

Preoperative bile duct interventions facilitate an alteration of the bile duct microbiome [10, 12]. Bacterobilia is associated with higher rates of septic complications, especially SSI [10]. Enterobacter and Enterococcus species, which are frequently detected after interventions involving the biliary tract, often show high resistance to 2nd generation cephalosporins. Previous publications show that targeted perioperative antibiotic prophylaxis based on local resistance patterns result in lower rates of septic complications, including SSI. In addition, it has been described that extended intraoperative peritoneal lavage (EIPL) was associated with lower mortality and morbidity rates, and less pain in patients undergoing gastrectomy [11, 13]. Furthermore, EIPL reduced the incidence of septic complications and significantly increased the 5-year survival in this cohort.

The aim of the present study was to determine the effect of targeted antibiotic therapy adapted to local resistance patterns in combination with EIPL and selective decontamination of the digestive tract (SDD) on the incidence of superficial and intraabdominal SSI after PD.

## Methods

### Patient cohort

One hundred sixty-three patients underwent pylorus-preserving pancreatoduodenectomy (PPPD) or classic pancreatoduodenectomy (cPD) between May 2018 and May 2021 at the Department of Visceral, Thoracic and Vascular Surgery, University Hospital Carl Gustav Carus, TU Dresden. The study was approved by the local ethics committee (decision no. BO-EK-394072021)

All patient data were retrospectively collected from a prospective database. The data included age, sex, weight, height, body mass index (BMI), alcohol and nicotine abuse, comorbidities, indication for surgery, preoperative endoscopic interventions, neoadjuvant therapy, type and length of operation, blood loss, fistula risk score [14], length of hospital stay, length of ICU stay, histopathological data, morbidity and mortality rates. At our institution, we implemented perioperative selective decontamination of the digestive tract (SDD) in patients undergoing pancreatic surgery in January 2019.

### Surgical technique and enhanced anti-infective prophylaxis

The surgical technique of PD and critical steps of the operation have already been described elsewhere [15].

The skin was prepared using an electric clipper before the operation. The patients were categorized into two groups: the control group, patients received standard pAP consisting of cefuroxime in combination with metronidazole and was applied within thirty to sixty minutes prior to incision. Redosing of the cefuroxime is performed after 3 hours if the duration of operation exceeded this duration. The EAP group, patients received piperazillin/tazobactam according to our local resistance patterns [10] thirty to sixty minutes prior to incision and drugs were readministered 4 hours after the initial dose if the duration of the operation exceeded this duration. SSI prevention measures were performed according to the World Health Organization guidelines [16]. Surgical site handling: An alcohol-based antiseptic agent (Skinsept G [46 g ethanol, 27 g 2-propanol, hydrogen peroxide 30% povidone, ponceau 4R (E124), yelloworange S (E110), purified water and Skinsept F [70 g 2-propanol, 0.5 g chlorhexidine [D-gluconate], 1.5 g hydrogen-peroxide 30%, purified water [Ecolab Health care, Monheim am Rhein, Germany]) was used three times for skin preparation before incision. Abdominal drapes protected wounds intraoperatively. After transection of the bile, a bile duct swab is taken intraoperatively and examined microbiologically. There was no routine protocol for abdominal lavage, which was performed according to the discretion of the surgeon (n = 100). The EAP group received extended abdominal lavage with five litres of saline solution after reconstruction during PD. Intraabdominal drains were placed according to the discretion of the surgeon. After closure of the fascia (before skin closure), subcutaneous tissue was irrigated with povidone-iodine solution (B. Braun, Melsungen, Germany) followed by lavage with normal saline. SDD consisted of vancomycin, colistin, tobramycin and amphotericin B. Two doses were administered in the evening, and one dose was administered in the morning preoperatively.

### Outcomes

The primary outcome was the incidence of septic complications within 90 days after surgery. Secondary endpoints included 90-day morbidity and mortality after pancreatoduodenectomy. Surgical morbidity included postoperative pancreatic fistula (POPF), intraabdominal abscess, delayed gastric emptying (DGE), postpancreatectomy haemorrhage (PPH), anastomotic leakage, surgical site infection (SSI), pneumonia, urinary tract infection, pancreatitis, cholangitis and thrombosis. Postoperative complications were categorized using the Clavien‒Dindo Classification [17, 18]. PPH, DGE and POPF were defined and classified according to the latest international consensus definitions of the International Study Group of Pancreatic Surgery (ISGPS) [19–21]. SSI was defined according to the definitions proposed by the Centers for Disease Control and Prevention (CDC) [22].

### Statistical analysis

The statistical analysis was performed with the IBM SPSS Statistics program (version 28, SPSS Inc., Chicago, IL). Categorial variables were demonstrated as patient number and percentage of the patient cohort. Median and interquartile range were used for quantitative variables. Categorial and quantitative variables were analysed by using the Fisher’s exact test, unpaired t test and Mann‒Whitney U test in univariate analysis. Due to the significant influence of adapted perioperative antibiotic prophylaxis, EIPL and SDD on the incidence of SSI, we performed a univariate analysis on the predisposing factors of SSI. For statistically significant variables, we performed a stepwise multivariate regression analysis to identify independent risk factors. A value of *p* < 0.05 was considered statistically significant.

## Results

### Study cohort

One hundred sixty-three patients (65 females and 98 males) who underwent PD were included in our analysis. The median age was 67 years (interquartile range: 61.0–76.0) in both groups. There were no significant differences in age or sex between the control and intervention groups. The most frequent indication for PD was a malignant tumour (n = 80, 49%). The most frequently performed operation was pylorus-preserving PD (PPPD), accounting for 67% (n = 110). Pancreatic surgery was significantly more often combined with simultaneous portal vein resection in the control group (43% vs. 27%, p = 0.046). Fifty-three patients from the control group (53%) and fifty-eight patients from the intervention group (92%) received SDD (p = 0.001). A total of 52 of the included patients did not receive SDD preoperatively (32%), of which 25 patients developed a SSI (48%). In contrast, only 19% (n = 21) of patients who received SDD developed an SSI (p = 0.001). Furthermore, univariate analysis of the basic patient characteristics showed no significant difference in regard to comorbidities (Table 1). Eighty-three patients underwent endoscopic retrograde cholangiography (ERC) prior to the operation. Biliary drainage was performed more frequently in the control cohort (Table 1). In the entire cohort, 46 patients developed an SSI (28%). In the intervention group, the incidence of SSI was significantly lower than that in the control group (14% vs. 37%; p = 0.002). The incidences of POPF, leakage of the biliodigestive anastomosis, pneumonia, cholangitis, pancreatitis and reoperation did not differ significantly between the two groups (Table 2). Interestingly, the risks of developing gastrointestinal bleeding (18% vs. 5%, p = 0.016), DGE (52% vs. 11%, p = 0.001) and urinary tract infection (24% vs. 8%, p = 0.011) were significantly lower in the intervention group (Table 3).

**Table 1:** Basic characteristics of patients who underwent PD

	Overall *n*=163 *(n* *(%)*)*	Control cohort *n*=100 *(n* *(%*))*	Cohort with adapted pAP and EIPL *n*=63 *(n* *(%)*)*	*P*- value
Age	67.0 (61.0-76.0)°	67.5 (61.3-76.8)°	67.0 (60.0-76.0)°	0.848
Sex ratio (male : female)	98 : 65	61 : 39	29 : 26	0.870
BMI (kg/m²)	25.1 (22.5-28.0)°	25.1 (22.5-28.0)°	25.4 (22.5-28.4)°	0.580
Comorbidities				
• Diabetes mellitus	53*(32.5)*	34 *(34.0)*	19*(30.2)*	0.732
• Hypertension	114*(69.9)*	74*(74.0)*	40*(63.5)*	0.165
• Nicotine abuse	46*(28.2)*	28 *(28.0)*	18(*28.6)*	1.000
• Alcohol abuse	39*(23.9)*	24*(24.0)*	15*(23.8)*	1.000
• Neoadjuvant therapy	30*(18.4)*	20*(20.0)*	10*(15.9)*	0.542
Tumour entity				
• Neoplasm pancreatic head	80 *(49.1)*	46*(46.0)*	34*(54.0)*	0.339
• Chronic pancreatitis	19*(11.7)*	10*(10.0)*	9*(14.3)*	0.457
• Other histology	64*(39.3)*	44*(44.0)*	20*(31.7)*	0.140
ERCP	83*(50.9)*	56*(56.0)*	27*(42.9)*	0.107
•Biliary drainage	77*(47.2)*	53*(53.0)*	24*(38.1)*	0.077

*B﻿MI* body mass index, *ERCP* endoscopic retrograde cholangiopancreatography, *pAP* perioperative antibiotic prophylaxis. *Data are presented as n (%), if not indicated otherwise. °Data are presented as the median (IQR)

**Table 2:** Perioperative characteristics of patients who underwent pancreatoduodenectomy

	Overall *n*=163 *(n* *(%)*)*	Control cohort *n*=100 *(n* *(%*))*	Cohort with adapted pAP and EIPL *n*=63 *(n* *(%)*)*	*P*- Value
Type of operation				
• PPPD	110 (*67.5)*	71 (*71.0)*	39 *(61.9)*	
• cPD	53 *(32.5)*	29 *(29.0)*	24 *(38.1)*	0.303
Operating time [minutes]	388 (328.0-460.0)°	405.5 (327.3-482,3)°	370.0 (330.0-432.0)°	0.219
Blood loss during operation [litre]	0.5 (0.4-0.8)°	0.6 (0.4-0.8)°	0.5 (0.3-0.7)°	0.128
Portal vein resection	60 *(36.8)*	43 (*43.0)*	17 *(27.0)*	0.046
Arterial resection	9 *(5,5)*	7 (*7,0)*	2 *(3,2)*	0,484
Multivisceral resection	15 *(9.2)*	10 *(10.0)*	5 *(7.9)*	0.784
FRS				
• Low-risk	39 *(23.9)*	26 *(26.0)*	13 *(20.6)*	0.347
• Intermediate- risk	104 (*63.8)*	59 *(59.0)*	45 *(71.4)*	0.379
• High-risk	10 *(6.1)*	6 (*6.0)*	4 *(6.3)*	1.000

*PPPD* pylorus-preserving pancreaticoduodenectomy, *cPD* conventional pancreaticoduodenectomy, *FRS* fistula risk score, *pAP* perioperative antibiotic prophylaxis. *Data are presented as n (%), if not indicated otherwise. °Data are presented as the median (IQR)

**Table 3:** Perioperative morbidity and mortality

	Overall *n*=163 *(n* *(%)*)*	Control cohort *n*=100 *(n* *(%*))*	Cohort with adapted pAP and EIPL *n*=63 *(n* *(%)*)*	*P*- Value
POPF	43 (*26.4)*	26 *(26.0)*	17 *(27.0)*	1.000
• BL	1 *(2.3)*	1 *(1.0)*	0 *(0.0)*	1.000
• Grade B	23 *(14.1)*	11 *(11.0)*	12 *(19.1)*	0.170
• Grade C	19 *(11.7)*	14 *(14.0)*	5 (*7.9)*	0.319
PPH	50 (*30.7)*	38 *(38.0)*	12 *(19.1)*	
• Gastrointestinal haemorrhage	21 *(42.0)*	18 *(18.0)*	3 *(4.8)*	0.016
• Intraabdominal bleeding	29 *(58.0)*	20 (*20.0)*	9 (*14.3)*	0.405
DGE	59 *(36.2)*	52 (*52.0)*	7 *(11.1)*	<0.001
• Grad A	18 *(30.5)*	13 *(13.0)*	5 *(7.9)*	0.443
• Grad B	20 *(33.9)*	19 *(19.0)*	1 *(1.6)*	<0.001
• Grad C	21 *(35.6)*	20 *(20.0)*	1 *(1.6)*	<0.001
Anastomotic leakage				
Hepaticojejunostomy	7 *(4.3)*	3 *(3.0)*	4 *(6.4)*	0.431
SSI	46 *(28.2)*	37 *(37.0)*	9 *(14.3)*	0.002
• Conservative procedure	9 (*19.6)*	9 *(9.0)*	0 *(0.0)*	
• Wound healing in secondary intention	25 (*54.3)*	18 (*18.0)*	7 (*11.1)*	
• Negative pressure wound therapy	5 *(10.9)*	5 *(5.0)*	0 *(0.0)*	
• Secondary wound closure	2 *(4.3)*	1 *(1.0)*	1 *(1.6)*	
• Reoperation	5 *(10.9)*	4 (*4.0)*	1 *(1.6)*	
Urinary tract infection	29 *(17.8)*	24 *(24.0)*	5 *(7.9)*	0.011
Pneumonia	11 *(6.7)*	9 (*9.0)*	2 *(3.2)*	0.206
Cholangitis	3 *(1.8)*	3 *(3.0)*	0 *(0)*	0.284
Pancreatitis	11 *(6.7)*	9 *(9.0)*	2 *(3.2)*	0.206
Reoperation	40 (*24.5)*	27 *(27.0)*	13 *(20.6)*	0.455
Length of hospital stay [days]	17 (12.0-25.0)	16 (12.0-27.0)°	17 (11.0-25.0)	0.492
Clavien‒Dindo Classification				
• > Grade 2 at 30d	80 *(49.1)*	50 *(50.0)*	30 *(47.6)*	0.872
• >Grade 2 at 60d	86 (*52.8)*	55 (*55.0)*	31 *(49.2)*	0.521
• >Grade 2 at 90d	87 (*53.4)*	55 *(55.0)*	32 *(50.8)*	0.631
90-day mortality	15 *(9.2)*	10 *(10.0)*	5 *(7.9)*	0.784

*BL* biochemical leakage, *DGE* delayed gastric emptying, *pAP* perioperative antibiotic prophylaxis, *POPF* postoperative pancreatic fistula, *PPH* post pancreatectomy haemorrhage, *SSI* surgical site infection. *Data are presented as n (%), if not indicated otherwise. °Data are presented as the median (IQR)

Eighty patients (49%) experienced a CDC grade II or higher complication in the first 30 days after the operation. Comparing the two groups, no significant difference was found in the 30-, 60- or 90-day morbidity rate. The 90-day mortality rate was 9%. In the control group, the mortality rate was moderately increased (10% vs. 8%, p = 0.784).

### Predisposing factors for SSI

A univariate analysis was performed to identify the risk factors for the development of postoperative SSI. No relevant difference in sex, BMI, diabetes, nicotine or alcohol abuse, diagnosis, neoadjuvant therapy, or preoperative biliary drainage was observed between the groups. Of the 46 patients who developed an SSI, 33 were older than 67 years. Patients older than 67 years had a significantly increased incidence of SSI (50% vs. 72%, p = 0.015). Similarly, arterial hypertension was associated with a significantly higher incidence of SSI (65 vs. 83%, p = 0.036). Furthermore, the group of patients with an SSI had significantly higher intraoperative blood loss (0.664 l [0.388–0.725] vs. 0.811 l [0.500-1.075], p = 0.048). The duration of the operation was prolonged in the group of patients who developed a SSI but the difference failed to reach statistical significance (379 min [329.0-449.5] vs. 413.5 min [326.0-515.8], p = 0.268). A total of 111 patients (68%) received SDD preoperatively. Only twenty-one of the treated patients (19%) developed an SSI compared to 48% (25/52 patients) who did not undergo SDD (Table 4). A POPF grade C occurred in one-fifth of the patients (20%) who developed an SSI (p = 0.060). Patients with an SSI were significantly more likely to develop DGE (57% vs. 28%, p = 0.001). Nearly one-third of the patients with an SSI developed postoperative haemorrhage. Consequently, SSIs were significantly associated with PPH (p = 0.003) and intraluminal bleeding (p = 0.001). Furthermore, we found a significant association between SSI and pneumonia (p = 0.002).

**Table 4:** Univariate analysis of risk factors for SSI

	Overall *n* = 163 *(n (%)*)*	Cohort without SSI *n* = 117 *(n (%)*)*	Cohort with SSI *n* = 46 (*n (%)*)*	*P*- Value
Cohorts				
•Control cohort	100 (*61.3)*	63 *(53.8)*	37 *(80.4)*	**0.002**
• EAP Cohort	63 *(38.7)*	54 *(46.2)*	9 *(19.6)*	
Sex				
• Female	65 (*39.9)*	48 *(41.0)*	17 *(37.0)*	0.723
• Male	98 *(60.1)*	69 *(59.0)*	29 *(63.0)*	
Age [years]				
• < 67	71 (*43.6)*	58 *(49.6)*	13 *(28.3)*	**0.015**
• > 67	92 *(56.4)*	59 *(50.4)*	33 *(71.7)*	
BMI [kg/m²]				
• < 25	80 *(49.1)*	61 *(52.1)*	19 *(41.3)*	0.228
• ≥ 25	83 *(50.9)*	56 *(47.9)*	27 *(58.7)*	
Comorbidities				
• Hypertension	114 *(69.9)*	76 *(65.0)*	38 *(82.6)*	**0.036**
• Diabetes mellitus	53 *(32.5)*	34 *(29.1)*	19 *(41.3)*	0.142
• Nicotine abuse	46 *(28.2)*	33 *(28.2)*	13 *(28.3)*	1.000
• Alcohol abuse	39 *(23.9)*	30 (*25.6)*	9 *(19.6)*	0.541
Preoperative Intervention				
• Neoadjuvant therapy	30 *(18.4)*	22 *(18.8)*	8 *(17.4)*	1.000
• Biliary drainage	77 *(47.2)*	54 *(46.2)*	23 *(50.0)*	0.728
• SDD	111 *(68.1)*	90 *(76.9)*	21 *(45.7)*	**0.001**
Entity				
• Benign	28 *(17.2)*	21 *(17.9)*	7 *(15.2)*	0.819
• Malignant	135 *(82.8)*	96 *(82.1)*	39 *(84.8)*	
Entity				
• Neoplasm pancreatic head	80 *(49.1)*	55 *(47.0)*	25 *(54.3)*	0.487
• Pancreatitis	19 *(11.7)*	15 *(12.8)*	4 *(8.7)*	0.592
• Others	64 *(39.3)*	47 *(40.2)*	17 *(37.0)*	0.726
Type of operation				
• PPPD	110 *(67.5)*	83 *(70.9)*	27 *(58.7)*	0.142
• cPD	53 *(32.5)*	34 *(29.1)*	19 *(41.3)*	
• Portal vein resection	60 *(36.8)*	41 *(35.0)*	19 *(41.3)*	0.475
• Arterial resection	9 *(5.5)*	7 *(6.0)*	2 *(4.3)*	1.000
•Multivisceral resection	15 *(9.2)*	11 *(9.4)*	4 *(8.7)*	1.000
Operating time [minute]	388 (328.0-460.0)°	379 (329.0-449.5)°	413,5 (326.0-515.8)°	0.268
Blood loss during operation [litre]	0.500 (0.400–0.800)°	0.664 (0.388–0.725)°	0.811 (0.500-1.075)°	**0.048**
FRS				
• Low-risk	39 *(25.5)*	28 *(25.5)*	11 *(25.6)*	1.000
• Intermediate-risk	104 *(68.0)*	75 *(68.2)*	29 *(67.4)*	1.000
• High-risk	10 *(6.5)*	7 *(6.4)*	3 *(7.0)*	1.000
Length of hospital stay [days]	17 (12.0–25.0)°	15 (11.0-22.5)°	20.5 (15.0-35.75)°	**0.001**
Length of ICU stay [days]	2 (1.0–6.0)°	1 (1.0–3.0)°	3 (1.0–7.0)°	**0.001**
Morbidity				
POPF	43 *(26.4)*	28 *(23.9)*	15 *(32.6)*	0.323
** • **BL	1 *(0.6)*	1 *(0.9)*	0 *(0.0)*	1.000
• POPF grade B	23 *(14.1)*	17 *(14.5)*	6 *(13.0)*	1.000
• POPF grade C	19 *(11.7)*	10 *(8.5)*	9 *(19.6)*	0.060
DGE	59 *(36.2)*	33 *(28.2)*	26 *(56.5)*	**0.001**
• Grade A	18 *(11.0)*	13 *(11.1)*	5 *(10.9)*	1.000
• Grade B	20 *(12.3)*	11 *(9.4)*	9 *(19.6)*	0.109
• Grade C	21 *(12.9)*	9 *(7.7)*	12 *(26.1)*	**0.003**
PPH				
• Gastrointestinal haemorrhage	21 *(12.9)*	8 *(6.8)*	13 *(28.3)*	**0.001**
• Intraabdominal bleeding	29 *(17.8)*	14 *(12.0)*	15 *(32.6)*	**0.003**
Anastomotic leakage				
• Hepaticojejunostomy	7 *(4.3)*	3 *(2.6)*	4 *(8.7)*	0.099
Urinary tract infection	29 *(17.8)*	22 *(18.8)*	7 *(15.2)*	0.656
Pneumonia	11 *(6.7)*	3 *(2.6)*	8 *(17.4)*	**0.002**
Pancreatitis	11 *(6.7)*	6 *(5.1)*	5 *(10.9)*	0.295
Cholangitis	3 *(1.8)*	1 *(0.9)*	2 *(4.3)*	0.192
DVT	5 *(3.1)*	2 *(1.7)*	3 *(6.5)*	0.137
PE	13 *(8.0)*	8 *(6.8)*	5 *(10.9)*	0.520
Clavien‒Dindo Classification				
• ≤ Grade 2 at 30d	83 *(50.9)*	66 *(56.4)*	17 *(37.0)*	**0.036**
• ≥ Grade 3 at 30d	80 *(49.1)*	51 *(43.6)*	29 *(63.0)*	
• ≤ Grade 2 at 60d	77 *(47.2)*	62 *(53.0)*	15 *(32.6)*	**0.023**
• ≥ Grade 3 at 60d	86 *(52.8)*	55 *(47.0)*	31 *(67.4)*	
• ≤ Grade 2 at 90d	76 *(46.6)*	61 *(52.1)*	15 *(32.6)*	**0.036**
• ≥ Grade 3 at 90d	87 *(53.4)*	56 *(47.9)*	31 *(67.4)*	
90-day mortality	15 *(9.2)*	8 *(6.8)*	7 *(15.2)*	0.130

*BMI *body mass index, *DGE *delayed gastric emptying, *DVT *deep vein thrombosis, *FRS *fistula risk score, *pAP *perioperative antibiotic prophylaxis, *PE *pulmonary embolism, *POPF *postoperative pancreatic fistula, *PPH *post pancreatectomy haemorrhage, *SDD * selective decontamination of the digestive tract, *SSI *surgical site infection. *Data are presented as n (%), if not indicated otherwise. °Data are presented as the median (IQR)

The multivariate analysis revealed that targeted antibiotic therapy combined with EIPL and SDD significantly reduced the incidence of SSI (OR 0.374, 95% CI 0.149–0.935, p = 0.035). In contrast, advanced age > 67 years (OR 2.607, 95% CI 1.107–6.142, p = 0.028) was identified as a significant risk factor for the development of SSI after PD (Table 5).

**Table 5:** Multivariate analysis of risk factors for SSI

	Univariate analysis	Multivariate analysis		
Parameters	Unadjusted incident rate ratio (95% CI)	*P*- Value	Adjusted incident rate ratio (95% CI)	*P*- Value
EAP Cohort	0.284 (0.126-0.641)	0.002	0.374 (0.149-0.935)	0.035
Age	2.494 (1.194-5.215)	0.015	2.607 (1.107-6.142)	0.028
Hypertension	2.562 (1.093-6.006)	0.03	1.999 (0.767-5.208)	0.156
Blood loss during operation	1.694 (1.006-2.852)	0.047	1.537 (0.893-2.645)	0.121
POPF grade C	2.603 (0.982-6.900)	0.055	2.563 (0.785-8.368)	0.119
DGE	3.309 (1.629-6.721)	<0.001	1.412 (0.606-3.291)	0.425

*SSI* surgical site infection, *POPF* postoperative pancreatic fistula, *DGE* delayed gastric emptying, *OR* odds ratio

### Outcome of patients with surgical site infection

Patients who developed a SSI had a significantly prolonged intensive care and hospital stay (15 days (11.0-22.5) vs. 20.5 days (15.0-35.8), p = 0.001). In addition, the length of postoperative stay in the intermediate care unit was significantly longer than that of patients without an SSI (1 day (1.0–3.0) vs. 3 days (1.0–7.0), p = 0.001). The 30-, 60-, and 90-day morbidity rates were significantly higher in the group with SSI. A total of 29 patients (63%) with SSIs had complications greater than grade 2 according to the CDC, while only 51 (44%) patients without SSIs had complications greater than the CDC II (p = 0.036). Likewise, DGE, PPH, and pneumonia occurred significantly more frequently in patients with SSIs (Table 4). There was no significant difference in mortality between the two groups (6.8% vs. 15.2%, p = 0.130).

### Impact of EIPL and SDD on the development of SSI

Altogether, 24 (51%) of 47 patients who did not receive EIPL(-) nor SDD(-) developed an SSI. In contrast, of 58 patients who received SDD(+) and EIPL(+), only 8 (13%) developed an SSI. The subgroup that did not receive EIPL(-) nor SDD(-) had a higher risk of SSI than those who received SDD (p = 0.035). Patients who did not receive EIPL(-) or SDD(-) had significantly more SSIs than those who received EIPL(+) and SDD(+) (p = 0.001). Thus, patients who received SDD(+) preoperatively had a significantly lower risk of developing SSI. Extended peritoneal lavage did not significantly affect the incidence of SSI (Tables 6 and 7).

**Table 6:** Influence of EIPL and SDD on SSI

	Overall *n* = 163 (n (%))*	SSI n = 46 (n (%))*	No SSI *n* = 117 (n (%))*	
EIPL (-), SDD (-)	47 (*28.8)*	24 *(51.1)*	23 *(48.9)*	
EIPL (-), SDD (+)	53 (*32.5)*	13 *(24.5)*	40 *(75.5)*	
EIPL (+), SDD (-)	5 (*3.1)*	1 *(20.0)*	4 *(80.0)*	
EIPL (+), SDD (+)	58 *(35.6)*	8 *(13.8)*	50 *(86.2)*	

*EIPL *extended intraoperative peritoneal lavage, *SDD *selective decontamination of the digestive tract, *SSI *surgical site infection. *Data are presented as n (%), if not indicated otherwise

**Table 7:** Binary logistic regression analysis of EIPL and SDD on SSI

	SDD	EIPL	*P*- Value	
EIPL (-), SDD (-)	+	-	**0.035**	
EIPL (-), SDD (-)	-	+	0.604	
EIPL (-), SDD (-)	+	+	**0.001**	
EIPL (-), SDD (+)	+	+	0.996	
EIPL (-), SDD (+)	+	+	0.483	
EIPL (+), SDD (-)	+	+	0.982	

*EIPL *extended intraoperative peritoneal lavage, *SDD *selective decontamination of the digestive tract, *SSI *surgical site infection

## Discussion

The incidence of surgical site infections (SSI) in patients undergoing pancreatic surgery is high and is associated with prolonged hospital stay, increased rates of reoperation, and increased mortality, all of which affect patient prognosis [3, 8].

In a previous study, we investigated the germ spectrum in the intraoperative biliary tract smear and the corresponding resistance patterns in our institution. We demonstrated that the most frequently detected germs were Enterobacter and Enterococcus species, which were resistant to common perioperative antibiotic prophylaxis (pAP, cefuroxime and metronidazole). As a result, we changed the pAP regimen to piperacillin and tazobactam according to local resistance patterns. In the present study, we investigated the effect of an EAP protocol, i.e., targeted antibiotic prophylaxis in combination with EIPL and selective decontamination of the digestive tract (SDD), on septic complications, especially SSI, in patients undergoing PD. This protocol was implemented as a novel standard to reduce the incidence of SSI and was prospectively documented.

The incidence of SSI in the present study was 28%, which is similar to that reported in previous studies [8–11]. Recent results suggest that the incidence of SSI can be reduced by adjusting pAP according to local resistance patterns. For example, the results of a nonrandomized study demonstrated that adjusting pAP can significantly reduce the incidence of SSI in patients undergoing PD (11% vs. 2%, p = 0.001) [23]. Other studies confirmed these results [12, 24]. The incidence of SSI was also significantly reduced by shifting the pAP to piperacillin and tazobactam (p = 0.001) [12]. Our data confirm these results; patients who received a targeted pAP had a significantly lower incidence of SSI after PD (37% vs. 14%, p = 0.002).

EIPL seems to be a promising technique to reduce the risk of postoperative septic complications [11, 13]. Transection of the bile duct during PD can lead to intraoperative contamination of the abdominal cavity and wound by bacterial colonized bile, thereby facilitating the development of septic complications [25]. EIPL can significantly reduce the amount of pancreatic and bile juice and wound exudate and clean the peritoneal cavity. A study of patients undergoing gastrectomy showed reductions in the incidences of postoperative complications such as intraabdominal abscesses, ileus, and SSI with surgery combined with EIPL compared with surgery alone [13]. In contrast, the present study did not confirm any significant association between EIPL and SSI.

Interestingly, patients who received EIPL in the present analysis had a significantly lower incidence of DGE. Hypothetically, EIPL may significantly reduce contamination in the abdominal cavity, thereby preventing the occurrence of DGE.

The data of several randomized studies show a reduction in the incidences of SSI and anastomotic leakage due to SDD in the perioperative period of colorectal surgery. Therefore, SDD has been firmly implemented in patients undergoing colorectal surgery [2, 3, 5, 6, 26]. In addition, the incidence of anastomotic leakage has also been sustainably reduced in patients undergoing oesophageal and gastric surgery. There are few studies that have investigated the impact on septic complications and anastomotic leakage in patients undergoing pancreatic surgery. The present data demonstrate that patients who received EAP, including pAP, SDD, and EIPL, had a significantly lower incidence of SSI. Further subgroup analyses suggest that the incidence of SSI may be affected by SDD (p = 0.035) but not by EIPL.

Advanced age, male sex, arterial hypertension, malnutrition, obesity, and immunosuppression are known risk factors for the development of SSI [8, 27, 28]. The present results show significantly higher rates of SSI in patients older than 67 years and in patients with arterial hypertension. The data in the present study did not show the same results in relation to sex, malnutrition, immunosuppression, or BMI. We did not investigate the impact of immunosuppression or malnutrition. Regarding sex and BMI, maybe a larger number of cases might have significantly changed the results. Similarly, longer operation time, higher intraoperative blood loss, and transfusions were associated with higher morbidity [28–30]. Patients with longer operation times were predisposed to develop SSI, although the difference was not statistically significant (p = 0.268). Furthermore, our data show a significant association between SSI and high intraoperative blood loss. Blood loss greater than half a litre was a significant risk factor for the development of SSI (p = 0.048).

### Limitations of the study

The present analysis has several limitations that should be considered when interpreting the results. Despite the limitations that our study was conducted retrospective at a single center and due to the small sample size, which may affect the statistical differences that are particularly relevant for the multivariate analyses of this manuscript, there is no other study to date on intensified EAP in patients undergoing pancreatic surgery. Furthermore, a potential selection bias can occur due to the small sample size. An EAP protocol was investigated, which differs in multiple items from the anti-infective management of the control group. The design did not allow the identification of pAP, EIPL, or SDD as a single measure to reduce SSI. There was an overlapping subcohort of patients who received SDD, which was addressed in subanalyses.

Regarding the literature, there appears to be resistance to standard perioperative antibiotic prophylaxis in pancreatic surgery. However, the local resistance patterns differ from hospital to hospital. According to the current literature, there is still no general recommendation for preoperative SDD in pancreatic surgery compared to e.g. colorectal surgery. Against this background, the external validity of our results are not generally applicable.

## Conclusion

The present results demonstrate that an EAP protocol including targeted infection prophylaxis with piperacillin and tazobactam according to local resistance patterns, EIPL, and preoperative SDD significantly reduced the incidence of SSI in patients after PD. Thus, EAP may be a radical approach to prevent SSI, but further studies with larger cohorts or randomized controlled trials are required to identify which of the different EAP measures has the most potential to lower the risk of SSI and which is potentially not effective. Furthermore, future studies should discover which patients benefit from EAP.

In recent years, minimally invasive surgical techniques have been increasingly implemented also in pancreatic surgery. These techniques are associated with a shorter length of stay without having a detrimental effect on morbidity, mortality and the oncological outcome. In our collective, we only evaluated open pancreaticoduodenectomies. However, in view of the above-mentioned development and the increasing use of minimally invasive techniques in pancreatic surgery, the influence of EAP should be investigated also in this context [31].

## Data Availability

No datasets were generated or analysed during the current study.
